# Changes in the composition of the RNA virome mark evolutionary transitions in green plants

**DOI:** 10.1186/s12915-016-0288-8

**Published:** 2016-08-15

**Authors:** Arcady Mushegian, Alexey Shipunov, Santiago F. Elena

**Affiliations:** 1 Division of Molecular and Cellular Biosciences, National Science Foundation, 4201 Wilson Boulevard, Arlington, VA 22230 USA; 2 Department of Biology, Minot State University, 500 University Avenue West, Minot, ND 58707 USA; 3 Instituto de Biología Molecular y Celular de Plantas, Consejo Superior de Investigaciones Científicas-Universidad Politécnica de Valencia, Ingeniero Fausto Elio s/n, 46022 València, Spain; 4 The Santa Fe Institute, 1399 Hyde Park Road, Santa Fe, NM 87501 USA

**Keywords:** Evolutionary transitions, In silico virus discovery, Metagenomics, Plant evolution, Transcriptome, Virus macroevolution

## Abstract

**Background:**

The known plant viruses mostly infect angiosperm hosts and have RNA or small DNA genomes. The only other lineage of green plants with a relatively well-studied virome, unicellular chlorophyte algae, is mostly infected by viruses with large DNA genomes. Thus RNA viruses and small DNA viruses seem to completely displace large DNA virus genomes in late branching angiosperms. To understand better the expansion of RNA viruses in the taxonomic span between algae and angiosperms, we analyzed the transcriptomes of 66 non-angiosperm plants characterized by the 1000 Plants Genomes Project.

**Results:**

We found homologs of virus RNA-dependent RNA polymerases in 28 non-angiosperm plant species, including algae, mosses, liverworts (*Marchantiophyta*), hornworts (*Anthocerotophyta*), lycophytes, a horsetail *Equisetum*, and gymnosperms. Polymerase genes in algae were most closely related to homologs from double-stranded RNA viruses leading latent or persistent lifestyles. Land plants, in addition, contained polymerases close to the homologs from single-stranded RNA viruses of angiosperms, capable of productive infection and systemic spread. For several polymerases, a cognate capsid protein was found in the same library. Another virus hallmark gene family, encoding the 30 K movement proteins, was found in lycophytes and monilophytes but not in mosses or algae.

**Conclusions:**

The broadened repertoire of RNA viruses suggests that colonization of land and growth in anatomical complexity in land plants coincided with the acquisition of novel sets of viruses with different strategies of infection and reproduction.

**Electronic supplementary material:**

The online version of this article (doi:10.1186/s12915-016-0288-8) contains supplementary material, which is available to authorized users.

## Background

The study of plant viruses since the early days has been dominated by the analysis of pathogens infecting crop plants. Isolation, characterization, and genome sequencing of plant viruses was later expanded to those viruses that have weeds or wild relatives of cultivated plants as their hosts. Viruses and virus-like replicons in non-agricultural habitats were in the meantime being discovered serendipitously, for example when symptoms suggestive of an infection were observed in nature, but it was only recently that technological developments – large-scale sequencing of cloned DNA at first, and next-generation sequencing in the last decade – allowed virologists to screen more systematically for the presence of known and novel viruses in plant populations of choice [1].

These efforts have provided us with a relatively good understanding of the general landscape of genomic diversity in viruses of angiosperm plants. This landscape appears to be completely devoid of viruses with large and/or linear DNA genomes, but includes DNA viruses with small, circular, single-stranded genomes; pararetroviruses that encapsidate double-stranded DNA and express their genome through an RNA intermediate; and a large variety of viruses with RNA genomes, comprising viruses whose virions encapsidate positive (+) or negative (–) sense single-stranded RNAs (ssRNA), double-stranded RNAs (dsRNA), or ambisense RNA. Sequence similarity analysis and phylogenetic studies have established profound, ancient relationships between each of these virus groups with viruses that infect fungi and animals; more recently, such studies have drawn the evolutionary links between these virus lineages and viruses infecting unicellular eukaryotes, bacteria, and archaea [2].

Despite this progress in our understanding of plant virus macroevolution, the sampling of the plant virome remains biased towards viruses of angiosperms, the most derived land plants. Interestingly, the only other subset of green plants with a relatively well-studied virome is represented by unicellular *Chlorophyta* [3, 4], which are thought to be a sister group to all land plants, as well as to the streptophyte algae. In a few chlorophytes, RNA virus-like replicons have also been characterized, such as partitivirus-like elements in the mitochondria and chloroplasts of *Bryopsis* [5]. On the whole, however, the profile of genome strategies in viruses infecting unicellular algae is very different from the viruses infecting flowering plants: the vast majority of viruses isolated from chlorophytes have large DNA genomes and belong to the phycodnavirus family [6], which, together with several other families infecting animals and various protists, constitute the apparently monophyletic group of nucleocytoplasmic large DNA viruses (NCLDV) [7, 8].

Thus, large DNA viruses dominate our view of virus genomic diversity in unicellular chlorophyte algae, whereas late branching angiosperms are thought to be infected mostly by RNA viruses and small DNA viruses. Only a few sequence reports exist for viruses infecting other green plants, including a (+)ssRNA virus in the multicellular, differentiated streptophyte *Chara australis* [9], a (+)ssRNA virus related to idaeoviruses that infects Japanese holly fern *Cyrtomium falcatum* [10], a nepovirus and a badnavirus from the gymnosperm *Cycas revoluta* [11, 12], and endogenous pararetroviruses expressed by genomes of ferns and gymnosperms [13]. In addition, transcriptionally inactive remnants of an NCLDV genome have been described in the genomic DNA of the green moss *Physcomitrella patens* and possibly also in the DNA contigs from the lycophyte *Selaginella moellendorffii* [14]. These examples notwithstanding, the virome of the whole phylogenetic expanse of green plant lineages that emerged after chlorophytes but before flowering plants has not been sufficiently characterized. The questions of the time and place of the switch between the predominantly-DNA and predominantly-RNA viromes in the evolution of land plants and their relatives, as well as the paths by which the RNA virome in higher plants has diversified to the extent observed today, remain unanswered.

The current consensus on the phylogeny of green plants, summarized in references [15] and [16] and in Table 1, considers chlorophytes and streptophytes to be two deep clades of green algae. The latter is thought to be the sister group of land plants (embryophytes), though considerable uncertainty remains as to which order within streptophyte algae is the closest to embryophytes [17]. Among the land plants, liverworts (*Marchantiophyta*) are thought to be the most basal branch, followed by the split of true mosses, and then of hornworts (*Anthocerotophyta*), which are likely the sister branch to vascular plants. The latter are divided into the lycophyte branch and a second branch that consists of seed plants and monilophytes (i.e., several small lineages including horsetails, as well as a large diverse fern lineage). Seed plants have two branches, angiosperms (flowering plants) and gymnosperms (including cycads, ginkgo, *Gnetales*, and conifers).

**Table 1:** RNA-dependent RNA polymerases in the land plant transcriptome libraries from the 1000 Plants Genomes Project

Phylum; Class; (group without taxonomic rank); Order	1000 Plants Genomes Project Library name and species	Double-stranded RNA viruses: phylogeny-based assignment or nearest match-based assignment	(−)RNA viruses: phylogeny-based assignment or nearest match-based assignment	(+) RNA viruses: phylogeny-based assignment or nearest match-based assignment
*Chlorophyta*; (no class assigned);(prasinophytes); *Pyramimonadales*	TNAW *Pyramimonas parkeae*	Basal group in picornavirus-like Clade 2, partitivirus		
*Chlorophyta*; *Chlorophyceae*; *Chlamydomonadales*	*Chlamydomonas acidophila*	Basal group in picornavirus-like Clade 2		
*Streptophyta*; *Zygnemophyceae*; *Desmidiales*	AEKF *Penium margaritaceum*	Unplaced picornavirus-like virus		
*Streptophyta*; *Zygnemophyceae*; *Zygnematales*	JOJQ *Cylindrocystis cushleckae*	New unplaced clade of dsRNA viruses		
*Streptophyta*; *Zygnemophyceae*; *Zygnematales*	HAOX *Spirogyra* sp.	New unplaced clade of dsRNA viruses		
*Streptophyta*; *Coleochaetophyceae*; *Coleochaetales*	DRGY *Chaetosphaeridium globosum*	New totivirus, sister branch to *Saccharomyces cerevisiae virus L-A*		
*Streptophyta*; *Coleochaetophyceae*; *Coleochaetales*	QPDY *Coleochaete irregularis*	Totivirus		
*Bryophyta*; *Marchantiopsida*; *Marchantiales*	TFYI *Marchantia emarginata*	Partitivirus		
*Bryophyta*; *Marchantiopsida*; *Marchantiales*	JPYU *Marchantia polymorpha*	Partitivirus		Potexvirus
*Bryophyta*; *Jungermanniopsida*; *Metzgeriales*	NRWZ *Metzgeria crassipilis*	Partitivirus		
*Bryophyta*; *Jungermanniopsida*; *Jungermanniales*	WZYK *Bazzania trilobata*	Partitivirus	Bunyavirus	Tobamovirus
*Bryophyta*; *Sphagnopsida*; *Sphagnales*	GOWD *Sphagnum lescurii*			Hepevirus, ourmiavirus, secovirus
*Bryophyta*; *Bryopsida*; *Hypnales*	JADL *Rhynchostegium serrulatum*		New clade in *Rhynchostegium* and *Hedwigia*	
*Bryophyta*; *Bryopsida*; *Hypnales*	QMWB *Anomodon attenuatus*			Hepevirus, tobamovirus
*Bryophyta*; *Bryopsida*; *Hypnales*	ZACW *Leucodon brachypus*			Potyvirus
*Bryophyta*; *Bryopsida*; *Hedwigiales*	YWNF *Hedwigia ciliata*		New clade in *Rhynchostegium* and *Hedwigia*	
*Bryophyta*; *Anthocerotopsida*; *Anthocerotales*	DXOU *Nothoceros aenigmaticus*	Totivirus?		
*Bryophyta*; *Anthocerotopsida*; *Anthocerotales*	TCBC *Nothoceros vincentianus*	New alphapartitivirus		Hepevirus
*Pteridophyta*; *Lycopodiopsida*; *Lycopodiales*	GAON *Huperzia squarrosa*	Partitivirus		
*Pteridophyta*; *Lycopodiopsida*; *Lycopodiales*	UPMJ *Pseudolycopodiella caroliniana*		Ophiovirus	Iflavirus, waikavirus
*Pteridophyta*; *Lycopodiopsida*; *Selaginellales*	ZZOL *Selaginella stauntoniana*			Hepevirus
*Pteridophyta*; *Equisetopsida*; *Equisetales*	CAPN *Equisetum diffusum*	Basal group in picornavirus-like Clade 2		
*Spermatophyta*; *Ginkgoopsida*; *Ginkgoales*	SGTW *Ginkgo biloba*	Partitivirus		
*Spermatophyta*; *Gnetopsida*; *Gnetales*	GTHK *Gnetum montanum*	Mitovirus		Marafivirus, secovirus, capillovirus
*Spermatophyta*; *Pinopsida*; *Podocarpales*	EGLZ *Prumnopitys andina*	Partitivirus		
*Spermatophyta*; *Pinopsida*; *Pinales*	PINU *Pinus taeda*			Cilevirus
*Spermatophyta*; *Pinopsida*; *Cupressales*	XMGP *Juniperus scopulorum*	Partitivirus		
*Spermatophyta*; *Pinopsida*; *Cupressales*	YFZK *Sciadopitys verticillata*		Varicosavirus	
Total number of new viruses		24 (7 assigned, 17 matched)	8 (4 assigned, 4 matched)	15 (1 assigned, 14 matched)

In this work, we used computational sequence analysis to detect several virus hallmark genes [18] in a collection of transcriptomes released in 2014–2015 by the 1000 Plants Genomes Project (1KP), which sampled species across the phylogenetic breadth of algae and green plants [15, 19]. We focused our attention on RNA viruses, even though transcripts from other kinds, such as ssDNA viruses, may also be found in these libraries. The 1KP data include transcriptome assemblies from 92 species of chlorophytes, streptophytes, and land plants. We selected the non-angiosperm plants, whose viromes are the least studied, and downloaded 66 transcriptomes form the 1KP Data Store hosted by the iPlant Collaborative [16]. As we show below, these libraries contain the evidence of diverse RNA virus-like replicons.

## Results

### Viral RNA-dependent RNA polymerases are pervasive in the transcriptomes of non-angiosperm plants

Viral RNA-dependent RNA polymerases (RdRP; EC 2.7.7.48) are a distinct group within the superfamily of nucleotidyltransferases with the catalytic right-hand palm domain. This superfamily includes several other kinds of DNA and RNA polymerases of viral and cellular origins, as well as more distantly related nucleotidyltransferases and nucleotidyl cyclases [20]. Viral RdRPs are themselves so diverse that establishing the monophyly of the RdRP from RNA viruses with different genome strategies has required sensitive computational approaches, such as matching of probabilistic models of previously defined protein families, aided by the arguments from comparative structural biology; nevertheless, RdRPs are closer to one another than to any other class of right-hand polymerases [21, 22].

Virus-like RdRP-encoding genes are not found in the DNA genomes of cellular organisms. The closest cellular homologs of RdRPs are the right-hand RNA-dependent DNA polymerases, or reverse transcriptases (RTs; EC 2.7.7.49) involved in propagation of retroviruses and retroelements, as well as their domesticated derivatives, such as telomerase and RNA splicing factor Prp8 in eukaryotes. Notwithstanding the sister relationship between RdRPs and RTs, these two groups of replication enzymes are clearly distinguishable in sequence similarity searches and with phylogenetic inference. Furthermore, viral RdRPs are not related to the cellular RdRPs involved in RNA interference, which possess a distinct configuration of conserved sequence elements and adopt a different double-psi barrel fold [23]. All this, together with the fact that every autonomously replicating RNA virus encodes for a palm-domain RdRP homolog, makes virus RdRPs uniquely suitable as phylogenetic markers and identifiers of RNA replicons in sequence databases.

We selected RdRP amino acid sequences representing every RNA virus family recognized by the International Committee for the Taxonomy of Viruses (ICTV), as well as the majority of the unclassified RNA viruses that are included in the ICTV database. These 80 protein queries were used to interrogate the joint nucleotide database of 66 green plant transcriptomes with the TBLASTN routine of the BLAST suite of programs [24]. The set of query proteins and the 1KP transcriptome libraries selected for analysis are given in Additional files 1 and 2, respectively. In addition, public nucleotide databases at the National Center for Biotechnology Information (NCBI), including the nucleotide NT database that contains the whole genome shotgun (WGS) division as well as the database of expressed sequence tags (dbEST), were searched using the same proteins as queries, and sequences homologous to virus RdRP were found in two algal species, the chlorophyte *Chlamydomonas acidophila* and the streptophyte *Penium margaritaceum*. The combined results of these searches are represented in Table 1, and fuller details on the identity of the contigs encoding these homologs are available as Additional file 3. Analysis of the data revealed that 1KP transcriptome libraries, even though constructed without regard to the possible presence of virus RNAs, in fact contain a wealth of information about the RNAs encoding novel RdRPs. In the following, we will to refer to these RNAs as virus-like replicons, or synonymously as viruses, even though replication of these molecules or their encapsidation have not yet been shown.

In total, RdRP homologs were discovered in the transcriptome libraries from 28 species, spanning the vast “virus desert” between green algae and angiosperms. In particular, RNA virus-like replicons are found in several classes of the chlorophyte algae – not only *Ulvophyceae* Ulvophyceae, where they have been noticed before [5, 25], but also *Chlorophyceae* and the prasinophyte *Pyramimonas parkeae*. Viruses were also found in streptophyte algae, including the first sightings in the representatives of classes *Coleochaetophyceae* and *Zygnemophyceae*. Among the land plants, RNA virus-like replicons are seen in two hornwort and four liverwort species, and also in several true mosses, from *Sphagnopsida* (*Sphagnum*), to more derived *Bryopsida* (*Anomodon*, *Hedwigia*, *Leucodon*, and *Rhynchostegium*). Furthermore, virus RNA polymerase-encoding replicons are seen in three species of lycophytes, in a horsetail *Equisetum*, and in several gymnosperms. All these putative viral RdRPs are novel – their sequences are not found in the nucleotide or protein databases at NCBI, except for the three ESTs mentioned above.

The lengths of the sequence fragments varied from 31 to 875 amino acid positions alignable with the closest database homolog (median 133), and the range of pairwise similarity to the nearest database homolog was from 19 % to 81 % (median 42 %). Despite this variation, all matches reported in Table 1 were statistically significant, and many included the characteristic conserved sequence domains – often, the iconic signature in the nucleotidyltransferase center, the GDD/SDD/GDN motif, that coordinates the metal ion cofactor and is directly involved in nucleotide addition [26–28].

We collected all cDNA fragments that showed statistically significant matches to virus RdRP protein sequences, and for each such fragment recorded the identity of its best match in the reverse BLASTX scan of the protein non-redundant (NR) database at NCBI. Out of 28 species in which we detected matches to any viral RdRP, 19 libraries encoded an RdRP most similar to a homolog from a virus with a dsRNA genome, five had virus replicons most similar to (–)ssRNA viruses, and 10 libraries contained encoded RdRP most closely related to a (+)ssRNA virus homolog (Table 1 and Additional file 3).

To further validate the virus origin of these contigs, we performed several tests. In the first test, we asked whether the similarity in a BLASTX search with default parameters of these 94 contigs against true viral sequences (protein NR database subset restricted by NCBI taxid:10239, “Viruses”) was significantly higher than the similarity to the best match annotated as non-viral plant sequences (NCBI taxid:33090, “Viridiplantae”). The distribution of *E*-values for the best matches against true viral sequences, although wide (range 0 to 4.83 · 10^−1^) was highly skewed towards a low value, with a median of 1.8 · 10^−33^. In contrast, the median of the distribution of *E*-values for the best match from a plant was 32 orders of magnitude higher, that is, 3.79 · 10^−1^ (range 1.08 · 10^−61^ to 9.61). Interestingly, all matches with *E*-values < 10^−10^ in this second search against “plant proteins only” corresponded either to proteins already classified as virus-like RdRPs (e.g., sequence BAA34783.1 from *Pyrus pyrifolia* or sequence BAB63954.1 from *Bryopsis cinicola*) or to unannotated proteins clearly related more closely to virus RdRPs than to proteins encoded by plant genomes. Moreover, such “plant” proteins typically came from cDNA sequencing projects, often explicitly targeted to recover virus RNA sequences, and did not appear to be represented in the genomic databases for the plant species they have been assigned to, suggesting that the vast majority of them are virus proteins annotated as host proteins. A paired-samples *t* test showed a highly significant difference between the two distributions of similarities (*P* = 5.7 · 10^−6^), and the statistic became even more extreme if all such putative RdRPs were removed from the list.

In another test, we used the 94 contigs to interrogate the entire NCBI nucleotide NT database in a TBLASTX search (with default parameters), and recorded the identity of the best matches for all the contigs. In 88 cases, the top match corresponded to a bona fide viral sequence, one case corresponded to a likely misannotated virus RdRP (the above sequence BAB63954.1 from *B. cinicola*), and the remaining five corresponded to uncharacterized proteins. Even allowing that all these six proteins are not confirmed to be of viral origin, the distribution of top matches is clearly dominated by viral sequences (binomial test, *P* < 10^−12^).

Finally, we tested whether our protocols were suited for recovery of the sequences that are truly encoded by plant genomes and are distantly related to virus replication enzymes of the palm-domain superfamily. For that purpose, several representative sequences from plant pararetrovirus RT domains were used as queries in the database searches of plant proteins or translated plant genomic DNA. When RTs from the pararetroviruses themselves, as well as from the integrated pararetrovirus-like sequences [13, 29], were excluded, all other highly significant matches were from the RTs encoded by the retroelements of the Ty3/copia class, the class of retroelements to which retrovirus and pararetrovirus RTs are most closely related [30].

The results of these tests strongly suggest that our protocols of sequence comparison identified true members of virus RdRP superfamily. Our protocols also provided a clear distinction between, on the one hand, RNA-encoded virus RdRPs, including those from cryptic viruses that are found in plant RNA databases but are not encoded by plant genomes, and, on the other hand, distantly related palm-domain RTs, whose gene sequences are found in RNA databases and also in DNA genome databases. Therefore, we conclude that the sequences listed in Table 1 are bona fide viral sequences, rather than unrecognized genome-encoded palm-domain sequences. Contextual information on other genes, in some cases found in the same libraries, argues the same (see below).

Simultaneously with this considerable evidence of virus RdRP genes – and, by implication, of RNA virus replicons in different green plants – this analysis reveals incompleteness, and perhaps a degree of inaccuracy, of the transcriptome assemblies in the 1KP project. The majority of predicted protein products similar to RdRPs were relatively short, accounting for only a part of the RdRP core domain; at the same time, the RdRP gene often was the longest and/or the only open reading frame on the entire assembled contig, and there was no evidence of nonsense mutations, out-of-frame mutations, or internal deletions within the coding region (data not shown). Therefore, despite the partial character of the matches, it seems unlikely that these RdRP-related sequences reside on aberrant transcripts from the integrated and pseudogenized DNA copies of virus genomes. Such fragments of integrated cDNA copies of non-retroid RNA viruses in any case may be rare in plants, as has been documented, for example, for grape genome [31]. Thus, it appears plausible that the protocols of transcriptome sequencing or assembly are producing only fragments of longer virus replicons. The finding of such a slew of partial RdRP sequences in the 1KP transcriptome dataset argues for additional efforts to recover full-length virus RNAs from green algae and vascular non-angiosperm plants.

### The most ubiquitous virus-like elements are related to the dsRNA partitiviruses

As seen from Table 1, most transcriptomes contain RdRPs whose nearest database homolog comes from a dsRNA virus. In unicellular and multicellular green algae, the RdRP-like sequences were exclusively of that origin. Often, the top match of an RNA virus was from *Partitiviridae*, a large family that includes viruses infecting plants, fungi, and protozoa. Partitiviruses typically possess two dsRNA genome segments, one encoding the RdRP and another encoding the capsid protein. Partitiviruses are transmitted by cell division, via plant seeds or pollen, fungal spores, or by hyphae anastomoses, but appear to lack the capacity to move from cell to cell via plasmodesmata, or by vasculature in plants. The vast majority of partitivirus infections are asymptomatic [32].

A taxonomy revision of partitiviruses, delineating four genera, has been proposed recently by the ICTV study group [33]. At a longer evolutionary span, all partitiviruses appear to form one clade (Clade 5) within the vast tribe of picorna-like viruses. Though that taxon has not been approved by ICTV, its monophyly is supported by the partially conserved gene content (i.e., versions of a characteristic five-gene array) and by the phylogenetic tree based on the alignment of RdRPs themselves [34]. Several groups of fungal dsRNA viruses belong to different clades within this tribe, supporting a hypothesis that dsRNA viruses may have evolved several times independently from (+)ssRNA viruses.

In order to place more precisely at least some of the partial RdRP sequences detected in our study onto the phylogeny of dsRNA viruses, we inferred a phylogenetic tree, utilizing the partitivirus RdRP dataset from Nibert et al. [33] and a much broader, but less densely sampled, picorna-like virus RdRP dataset from Koonin et al. [34]. The protein sequences in the two datasets were aligned, the quality of the alignment was verified by the consistency of the sequence motifs and by superimposition of the conserved secondary structure, and the novel RdRP sequences predicted by the conceptual translations of the 1KP RNAs were aligned to that master alignment. Many of the sequences recovered by our BLAST searches were short, and therefore unlikely to contain enough sites for phylogenetic inference; despite clear virus origin of such sequences, their phylogenetic placement must await longer alignments. Eight sequences, however, included substantial portions of the conserved core of RdRP sequence, in particular motifs A through D [35], and were retained. The final alignment, terminating right after the GDD motif (motif D), contained 146 aligned amino acid sites and 146 RdRP sequences (Additional file 4).

**Fig. 1:**
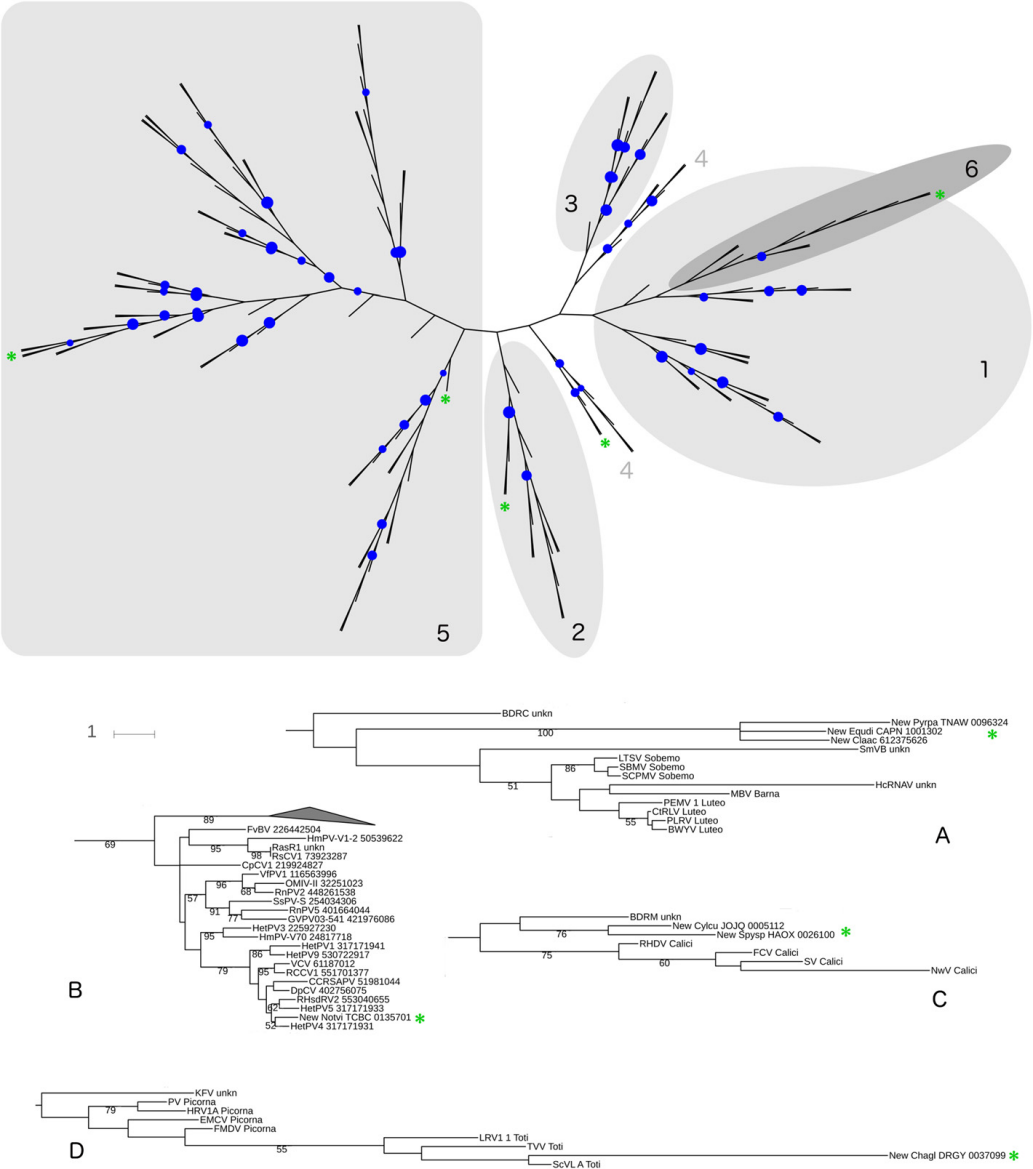
Maximum-likelihood tree of RNA-dependent RNA polymerases (RdRPs) from viruses of the picorna-like tribe. In the large tree (*top*), numbers indicate clades defined in Koonin et al. [34], and the bootstrap support is shown by *indigo dots* for all internal partitions where it was at least 60 %, with the dot size increasing as the values grow to 100 %. The branch lengths in the large tree are not to scale. *Green asterisks* indicate the position of new viruses. **a**–**d** The branch lengths of the subtrees are drawn to scale, with the bootstrap support shown for all partitions where it was higher than 50 %. Novel viruses are marked by *green asterisks*; their identifiers start with “New” and are followed by the ID of the 1000 Plants Genomes Project scaffold. Virus names, short descriptions of taxonomic position, and GenBank ID numbers are taken from Koonin et al. [34] and Nibert et al. [33], except that all sequence identifiers in the latter source referred to the nucleotide sequences and were replaced in this study by the IDs of their protein products that were actually aligned. **a** A clade of virus RdRPs from diverse hosts is a deep branch within the picornavirus-like Clade 2. **b** New alphapartitivirus from *Nothoceros vincentianus* (left-most branch in Clade 5 on *top*). **c** Two sequences from *Zygnematales* may be distantly related to picornavirus-like Clade 4. **d** A totivirus from *Chaetosphaeridium globosum*

We used the maximum-likelihood phylogenetic inference approach, implemented on the PhyML algorithm [36], to infer a phylogenetic tree of RdRPs from dsRNA and (+)ssRNA viruses of the picorna-like tribe. The support for the partitions of the tree was assessed using bootstrap resampling of the alignment. The resulting rootless tree, visualized with the aid of the iTOL server [37], is shown in Fig. 1 (the tree in Newick format is available in Additional file 5: source data for Fig. 1).

Five of the six clades proposed in Koonin et al. [34], as well as the division of partitiviruses into genera according to Nibert et al. [33], are visible in the tree, though the support for all clades was weaker than in the original studies, some of the basal branches had uncertain placement, Clade 6 (picornaviruses) was nested within Clade 1, and Clade 4 was split into separate deep-branching totivirus and calicivirus clades. Despite this loss of precision, which most likely occurred because we could use fewer aligned sites for phylogenetic inference than in the earlier studies, some phylogenetic assignments were possible for the new putative dsRNA viruses. First, there is a well-supported group of three viruses, two from chlorophytes and one from *Equisetum*, which appear to be a basal cluster close to Clade 2 (Fig. 1a). Second, the RdRP sequence from hornwort *Nothoceros vincentianus* clearly belongs to the genus *Alphapartitivirus* (Fig. 1b). Third, two sequences from putative viruses infecting *Zygnematales* are a likely to belong to Clade 4 (Fig. 1c). Fourth, a fast-evolving sequence from a streptophyte alga *Chaetomium globosum* is likely to be a totivirus related to the yeast virus L-A (Fig. 1d). These observations, together with the nearest-neighbor information for other dsRNA-related sequences (Table 1), suggest that the dsRNA virus-like replicons in the RNA libraries from the 1KP project tend to be closer to homologs infecting fungi and protists than to those infecting higher plants.

### ssRNA viruses rise with the transition from green algae to land plants

We found that RNA libraries of land plants also encode some RdRPs whose nearest database neighbors are viruses with dsRNA genomes. In addition, we identified other polymerases that were closer to viruses with ssRNA genomes of either positive or negative sense. Sometimes multiple RdRP homologs were found in a library from a single species, such as a liverwort *Bazzania trilobata*, which appears to harbor a dsRNA, a (+)ssRNA, and a (–)ssRNA virus (Table 1).

**Fig. 2:**
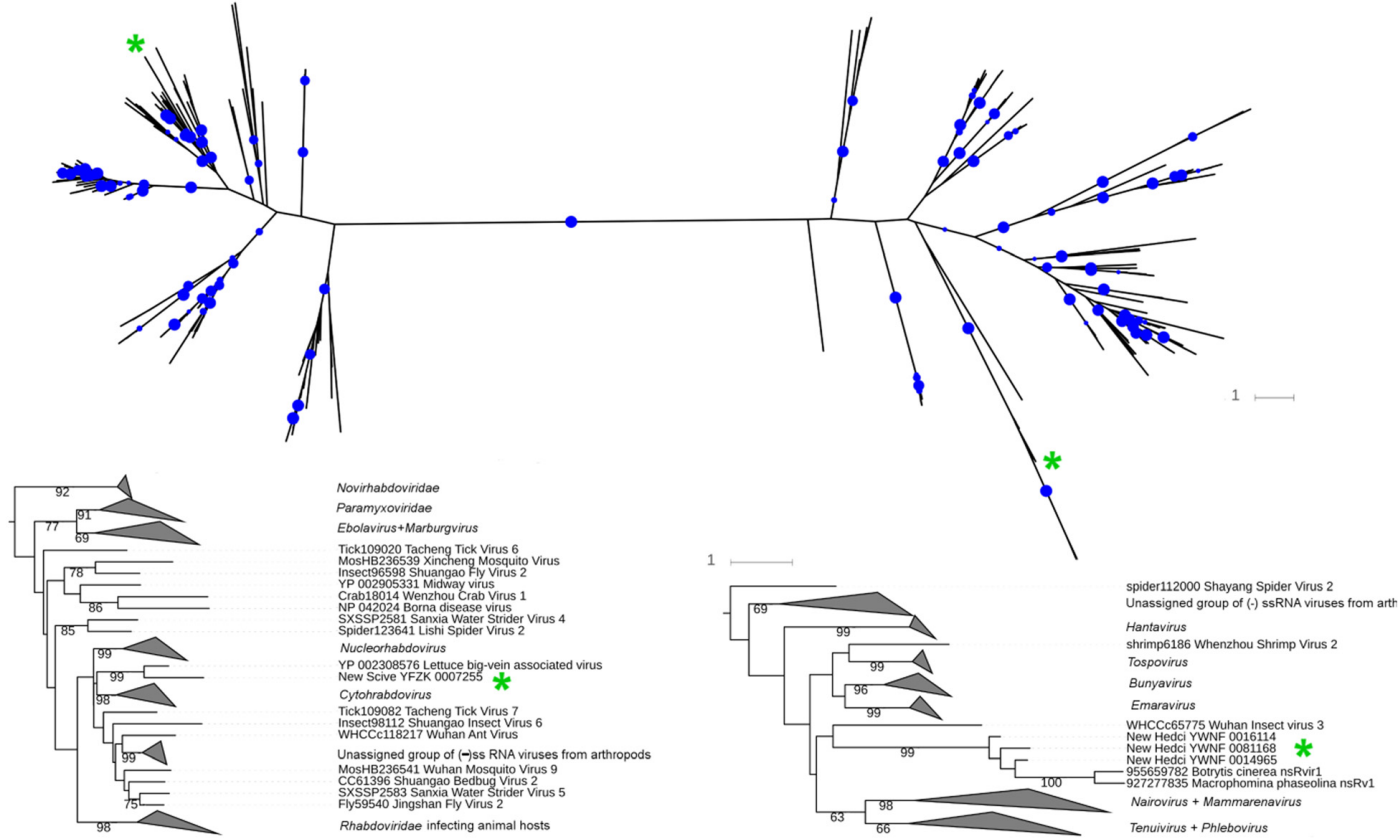
Maximum-likelihood tree of RNA-dependent RNA polymerases from (–)ssRNA viruses. In the tree on top, the *right-hand cluster* comprises Bunyaviridae, Arenaviridae, and related taxa, and the *left-hand cluster* comprises Mononegavirales and related taxa. The tree is drawn to scale, and the bootstrap support is shown by *indigo dots* for all internal partitions where it was at least 60 %, with the dot size increasing as the values grow to 100 %. Novel viruses are marked by *green asterisks*. The branch lengths of the subtrees are drawn to scale, the bootstrap values above 60 % are indicated by numbers, and novel viruses are marked by *green asterisks*; their identifiers start with “New” and are followed by the ID of the 1000 Plants Genomes Project scaffold. The clades corresponding to most genera are collapsed

As with putative dsRNA viruses, we attempted to phylogenetically assign at least some of the RdRP genes from ssRNA viruses. A large-scale sequencing and phylogenetic inference of viruses with (–)ssRNA genomes in arthropods has been completed recently, which revealed a substantial biodiversity, including at least one new family-level clade [38]. We took advantage of the sequence library compiled in that study, and aligned it together with the longest (–)ssRNA sequences from the 1KP libraries, as well as putative RdRPs from two recently identified (–)ssRNA viruses of fungi *Botrytis cinerea* and *Macrophomina phaseolina* (GenPept IDs 927277835 and 95565978). Phylogenetic inference was performed on the alignment of 219 sequences that contained 259 sites (Additional file 6). The resulting tree is shown in Fig. 2 (the Newick-formatted tree is available as Additional file 7: source data for Fig. 2). Upon examination of the tree topology, we were able to assign a putative virus infecting a gymnosperm *Sciadopitys verticillata* to the genus *Varicosavirus*. A group of closely related viruses in moss *Hedwigia ciliata* was assigned to a novel clade that appears to also include the aforementioned two RdRPs of fungal viruses; this group tentatively clusters with the polymerase of *Wuhan insect virus3*, though without statistical support. These viruses form one or two clades that are as deep as the adjoining genera, and may represent a novel virus genus or perhaps even a family. Other sequences, though quite clearly related to RdRPs with (–)RNA genomes, were too short to provide clear phylogenetic assignment, though partial sequences from the green moss *Rhynchostegium serrulatum* almost certainly belong to the same novel clade as its close homologs from *H. ciliata* (Table 1 and data not shown). We also expect that partial sequences in the liverwort *Bazzania trilobata* and in the lycophyte *Pseudolycopodiella caroliniana* will be assigned, respectively, to bunyaviruses and to ophioviruses after full-length sequencing.

**Fig. 3:**
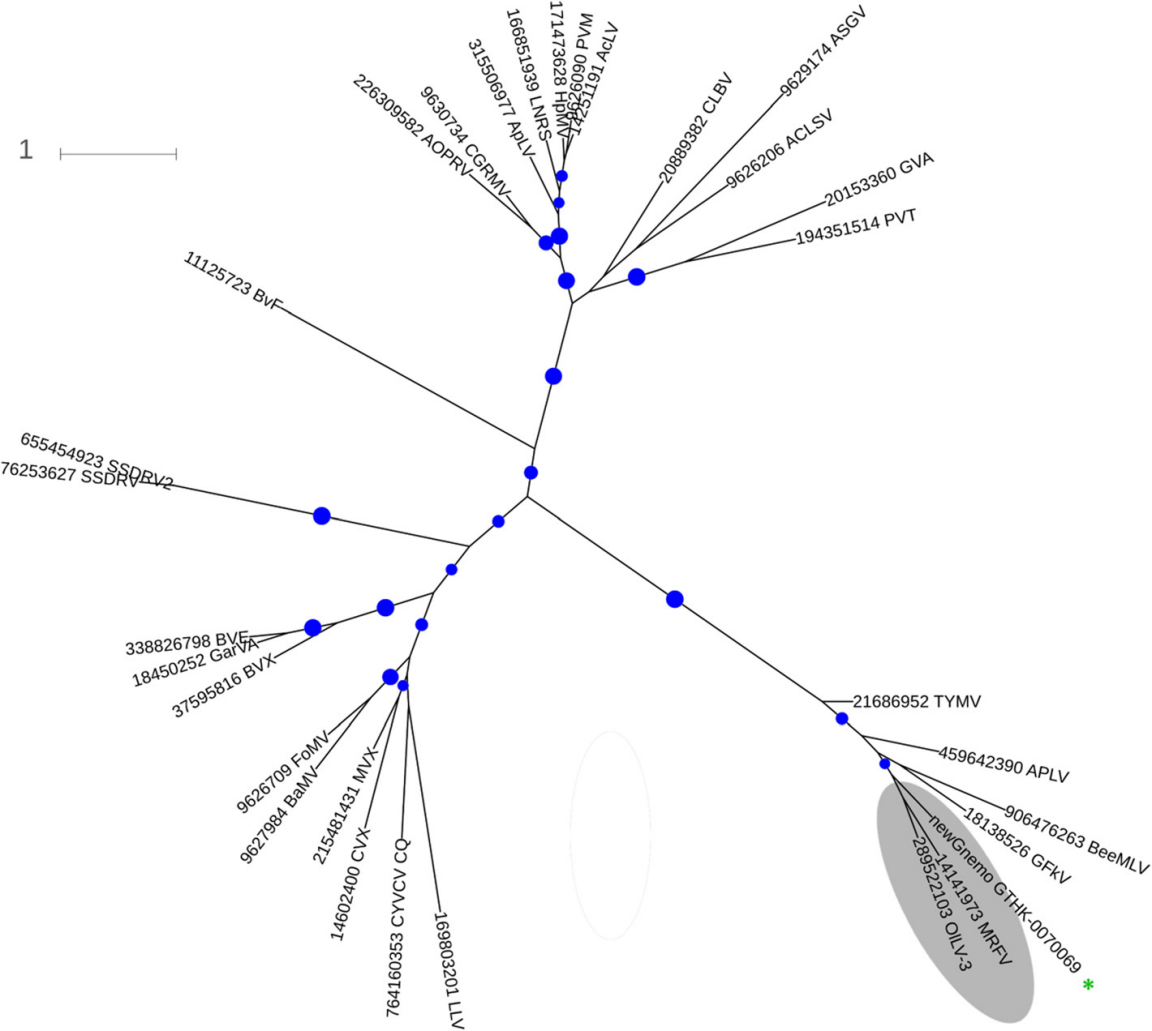
Maximum-likelihood tree of RNA-dependent RNA polymerases from the viruses in order *Tymovirales*. The bootstrap support values of more than 60 % are shown by *indigo dots* for the internal partitions. *Gray shade* indicates genus *Marafivirus* that includes a novel virus from *Gnetum* indicated by *green asterisk*

The picture was qualitatively similar for RdRPs from putative (+)ssRNA viruses: partial sequences were found in different clades of land plants, with the identity of the nearest database matches suggesting a variety of virus families and genera. We were able to assign a partial sequence from a gymnosperm *Gnetum montanum*, which contained a nearly complete RdRP domain, to genus *Marafivirus* (*Tymovirales*) (Fig. 3; alignment is available in Additional file 8; Newick-format tree is available in Additional file 9: source data for Fig. 3).

### RNAs encoding for other virus hallmark proteins are differentially distributed

We examined all cDNA fragments that coded for RdRP, and found that one of them, the scaffold from *Gnetum montanum* GTHK-0070069, also contained a nearly-complete open reading frame in the same strand, encoding a homolog of the marafivirus capsid protein. Prompted by this finding, we asked whether any of the libraries had sequences encoding for virus capsid proteins, another class of virus hallmark genes possessing spatial folds that are rare in cellular proteins [18]. We collected the sequences of representative structural proteins from the virus groups that were suggested by the RdRP nearest-match analysis and searched the 1KP libraries. Potential cognate capsid proteins were identified for some of the virus-like replicons described above, such as a fragment of an alphapartitivirus capsid protein in the hornwort *Nothoceros vincentianus*, capsids of unassigned partitiviruses in the lycophyte *Huperzia squarrosa* and the gymnosperm *Prumnopitys andina*, and an ophiovirus-like capsid protein in the lycophyte *Pseudolycopodiella caroliniana* (list of queries and matches is available as Additional file 10). Genomes of viruses from all these groups are segmented, and their capsid genes are expected to reside on different scaffolds than the RdRP gene. Hence, it is technically possible that in some of these cases not one but two related viruses are present in the same host.

Another class of plant virus hallmark proteins are movement proteins of the 30 K superfamily [13]. This superfamily is found in the genomes of different groups of plant ssRNA viruses, as well as in pararetroviruses and in bipartite geminiviruses with ssDNA genomes; it appears to be a stand-alone sequence superfamily with a putative all-beta fold. Plant genomes do not encode recognizable 30 K homologs, except for the pararetrovirus 30 K genes, which are found integrated, together with the rest of the virus, in the genomes of almost all angiosperms for which complete sequences are available. In an earlier work, we noted that sequences homologous to pararetrovirus 30 K genes were also found in the EST databases of gymnosperms and ferns, but neither among the ESTs nor in the completely sequenced genomes of the green moss *Physcomitrella patens* and the lycophyte *Selaginella moellendorffii* [13]. In this study, we searched the 1KP transcriptome libraries with representative 30 K-superfamily proteins and detected RNAs encoding pararetrovirus 30 K movement proteins in several gymnosperms, in a fern *Pteridium aquilinum*, and, interestingly, in two lycopods, *Pseudolycopodiella caroliniana* and *Dendrolycopodium obscurum* (the list of queries and matches is available in Additional file 11). The transcriptome of *P. caroliniana* furthermore contains distinct sequences related to the 30 K proteins of a (+)ssRNA capillovirus and of a (–)ssRNA ophiovirus. This extends the host range of the 30 K protein-encoding plant viruses to lycopods.

Finally, we asked whether the NCLDV core gene products [39] could be detected in any of the 1KP transcriptome libraries. Similarity searches detected such sequences, closely related to orthologs from phycodnaviruses, in the algae *Pyramimonas*, *Penium*, *Cylindrocystis*, and *Chaetosphaeridium*, whereas in land plants all statistically significant matches appear to belong to transcripts that are paralogous, but not orthologous, to NCLDV genes, as judged by reverse searches (data not shown). An earlier report of NCLDV DNA fragments integrated into the genome of the green moss *Physcomitrella* has concluded that similar fragments also were present in another related species of moss but were not transcribed in either of the two hosts [14]. On the other hand, the purported occurrence of two short NCLDV-derived integrated regions in the lycophyte *S. moellendorffii* [14] is not supported by phylogenetic analysis: the viral origin of one of these genes was dismissed in the original publication itself, whereas the remaining one, the homolog of 5′-3′ exoribonuclease Xrn, appears to be a deep, unresolved branch in the Xrn family tree, slightly closer to a sequence encoded by complete genome of the yeast *Wickerhamomyces ciferrii* than to the NCLDV homologs (Additional file 12). Thus, it is unclear whether NCLDV-derived genes, or even pseudogenes, occur in land plants beyond a subset of green mosses.

## Discussion

In this study, we have provided the evidence of diverse RNA plant virus-like replicons in non-angiosperm land plants and in their algal relatives, that is, throughout the plant kingdom. The sequence libraries analyzed in our work have not been targeted towards selective recovery of virus RNA; the sampling of plant species by the 1KP project was phylogenetically broad but not particularly dense, likely affecting the accuracy of tree reconstruction; virus genes with low similarity to the hallmark sequences were most likely missed in our searches; and most matches to virus genes that we detected were partial and resided on short cDNAs. Despite these concerns, and notwithstanding that the fern libraries drew a near-blank, we identified – as far as we know, for the first time – sequences homologous to various hallmark genes of RNA viruses in true mosses, hornworts (*Anthocerotophyta*),^1^ and liverworts (*Marchantiophyta*), in lycophytes, in a horsetail *Equisetum*, and in a broader range of green algae than before. Some of the live plants used as the source of RNA in the 1KP effort are likely not to be axenic, so it is possible that some of the virus replicons seen in our study in fact come not from the plant cells, but from tightly associated fungi or other eukaryotes that may be a part of the plant holobiont. This possibility requires further examination.

In the earlier studies (e.g., references [33, 34, 40]), the similarities between plant and fungal dsRNA viruses, often to the exclusion of animal dsRNA viruses, were interpreted mostly as the evidence of recent horizontal exchanges of viruses between multicellular plants and fungi, perhaps most likely parasitic or endophytic fungi closely associated with the plant hosts. This hypothesis is boosted by the recent reports of mycosomes, that is, fungal protoplasts capable of invading the plant cell [41], which may be likely vehicles for virus transfer. Our observations that the numerous RdRPs genes found in the RNA libraries from chlorophyte and streptophyte algae are also related to the homologs from fungal dsRNA viruses, especially when viewed together with the recent demonstration that mutualistic relationship between a chlorophyte and an ascomycete could be established experimentally [42], suggests that plant–fungal interactions and viral exchanges may in fact have a much longer history.

Various dsRNA viruses, all of which belong to the picorna-like tribe, have been found in four out of five supergroups of protists; it has been suggested that they evolved from their ancestors with ssRNA genomes in the “Big Bang” of picornavirus radiation in primitive eukaryotes [34]. Despite this postulated ancestral character of ssRNA viruses, very few ssRNA virus-like replicons have been characterized in green algae thus far ([9] and this study), whereas putative dsRNA viruses are found in multiple lineages of chlorophyte and streptophyte algae. It remains unclear whether specific ecological factors impose a constraint on the diversity of RNA viruses in unicellular eukaryotes, favoring dsRNA replicons over single-strand ones. Whatever this constraint might be, it is apparently relieved in multicellular eukaryotes, including all land plants: the burst in diversification of genome strategies in plant RNA viruses is seen in both moss and vascular plant lineages. Nothing is known about the manifestation of these virus infections in their hosts, though it is quite likely that the putative ssRNA viruses uncovered in this work are latent or asymptomatic, if only because symptomless host specimens are more likely to be chosen for cDNA library construction.

The common ancestor of mosses and vascular plants may have existed about 475 MYA, in the Ordovician [43]. Interestingly, this is the time when insect diversification was also taking place, and rapid evolution of land arthropods may have been a factor in the emergence of novel clades of RNA viruses, especially as recently documented for viruses with (–)ssRNA [38]. The relative closeness of some of the (–)ssRNA viruses from mosses to an orphan insect virus is compatible with the hypothesis of a secondary invasion of land plants with ssRNA viruses, mediated in part by insects [38]. On the other hand, the fact that the same clade includes viruses of fungi is also notable.

Yet another bias in the virus sequences shown in Table 1 is that they are dominated by viruses from the taxa whose full-length genomic or subgenomic mRNAs are polyadenylated at the 3′ end. This is to be expected, because the cDNA libraries in the 1KP effort were prepared from polyA+ RNA [44]. A putative tobamovirus-like replicon from the liverwort *Bazzania trilobata*, which is likely to carry a 3′ tRNA-like structure instead of the polyA, is one exception from this trend. Interestingly, the same trend appeared in a pilot bioinformatics study of virus-derived small RNAs [45], which used sRNA and miRNA libraries from various land plants [46]. Thus, targeted discovery of plant RNA virus replicons is needed to provide a more complete picture of the land plant virome. We feel, however, that the main conclusion, that is, the presence of dsRNA viruses in the ancestral lineages of green plants and later stepwise accrual of more diverse viruses, will remain unchanged, notwithstanding a likely sampling bias mentioned above.

Our analysis also revealed the existence of virus movement proteins from the 30 K superfamily in a lycopod virome. In contrast, RNA libraries from true mosses, hornworts, and liverworts showed a wide variety of RdRP gene products but did not contain their cognate, or indeed any, 30 K-superfamily proteins. Further analysis of RNA viromes from mosses and their relatives will show whether the 30 K proteins have established themselves throughout the plant viruses, or only among those viruses that infect hosts with the dominant sporophyte phase.

## Conclusions

We found that colonization of land and subsequent growth in anatomical complexity in plants coincided with the acquisition or expansion of novel sets of RNA viruses with different strategies of infection and reproduction. Evidence of various hallmark genes of RNA viruses have been found in mosses, hornworts, liverworts, lycophytes, horsetail, and in a broader range of green algae than before. Analysis of the phylogenetic affinities of the putative dsRNA and ssRNA replicons suggests that viruses associated with the evolutionarily more ancient groups, such as chlorophyte and streptophyte algae, tend to form deeper clades in the phylogenetic trees. Conversely, RNA viruses from land plants more often fall within the families or genera that have already been established by analysis of viruses infecting angiosperms and fungi.

## Methods

Sequence database searches were mostly performed in July 2015. The 80 protein queries were RdRP sequences representing every RNA virus family recognized by ICTV, as well as the majority of the RNA viruses that are included in the ICTV database but unclassified (Additional file 1). These 80 queries were used to interrogate the concatenated nucleotide database of 66 non-angiosperm green plant transcriptomes selected from the 1KP project [15, 19], which are listed in Additional file 2. The database consisted of 2,673,061 sequences with 1,019,254,703 total letters, including internal Ns. The nucleotide sequence databases (NT and dbEST) and the non-redundant protein sequence database (NR) at NCBI were also searched. All searches were done with the BLAST family of programs [24] with the SEG filter (-F option) typically set at false. The information on the RNA polymerase core domains from the Conserved Domains Database (CDD) database [47] was utilized to trim the sequences to include only the most conserved domains of RdRPs.

All moderately significant matches to RdRP proteins in the 66-species subset of the 1KP transcriptome database (BLAST *E*-value ≤ 0.005) were collected and verified by reverse BLASTP and BLASTX searches against the NCBI NR database. The protein alignments produced by reverse BLAST were inspected for the presence of conserved sequence motifs. In these reverse searches, > 91 % of the selected transcriptome library sequences showed statistically significant matches to the homologs from viral RdRP. The remaining sequences were either closely similar to an unrelated database protein sequence or without homologs in the database; those were not examined further. One sequence from the *Penium margaritaceum* library (scaffold AEKF-0068309) was identical to a fragment of RdRP of *Tobacco vein banding mosaic virus*, a potyvirus, at both the nucleotide and amino acid levels; this is the only instance of a likely library contamination that we saw in this study, and it was also removed from further analysis.

For phylogenetic inference, the partitivirus dataset from Nibert et al. [33] was downloaded from the publisher’s website, the “picorna-like” dataset from Koonin et al. [34] was generously provided by Dr Y. Wolf, and the (–)ssRNA virus dataset from Li et al. [38] was generously provided by Dr E.C. Holmes and Dr M. Shi. In all these cases, protein sequences were aligned using the MUSCLE program [48], first joining closely related sequences and then combining those alignments using the -profile option to obtain a master alignment. To obtain the master alignment of RdRPs from (–)ssRNA viruses, the HHPred server [49] was queried with the polymerase domain of *Sonchus yellow net virus* to build a profile Hidden Markov Model (profile-HMM) and to interrogate the profile-HMMs generated from the CDD; the automatically generated, highly scoring alignment between rhabdovirus and bunyavirus RdRP models was used as a guide to trim both ends of the long alignments of RdRP-containing multidomain proteins of mononegavirales and bunyavirales, and the two alignments were aligned together using the -profile option of the MUSCLE program. Orthomyxovirus replication enzymes were excluded from the analysis because they were much more distantly related to the plant virus sequences than the other virus clades (data not shown). The new sequences described in this study were first aligned to the protein family to which they showed the highest similarity in BLAST searches, and this information was used to remove the distal portions of the master alignment not shared with the partial sequences from the transcriptome library. The alignment of the marafivirus RdRP domain was also obtained using the MUSCLE program and checked against the alignment of multidomain replicases (J. Kreuze, pers. commun.) that was submitted to ICTV in 2006 as part of the proposal that led to establishing the order *Tymovirales* in 2009.

Phylogenetic inference was done using the PhyML server [36] with a Le and Gascuel (LG) substitution model that was selected automatically based on the data [50], other parameters estimated from the data, and 1000 bootstrap replicates performed in order to assess the support of the internal partitions in the tree. For the largest dataset of (–)ssRNA virus RdRP, we confirmed the LG model selection with the PhyML server, but utilized the E-Biothon supercomputing platform [51] for shorter computation times. The iTOL server [37] was employed for tree examination and visualization.

To reanalyze putative NCLDV sequences thought to be integrated in the lycopod genome, we could not use the data from t he original study (Maumus et al., [14]), which identified a 184-amino-acid EFJ26844 as the putative NCLDV-derived gene product, but indicated that their phylogenetic inference used a longer product reconstructed by screening for overlapping scaffolds, because the details of that reconstruction are not available. Therefore, we used another *Selaginella moellendorffii* gene product, the 298-amino-acid EFJ10074, which includes the complete sequence of EFJ26844 with only three mismatches. The closest 250 BLASTP matches to EFJ10074 were collected, and each group of sequences from one genus that had multiple homologs with more than 95 % identity in the same genus was replaced by one representative sequence; this mostly concerned yeast *Candida* and angiosperms from the core eudicots. The alignment and tree were constructed as described above.

## Additional files

Additional file 1: The library of viral RdRP domains used as queries in this study. (DOC 88 kb) Additional file 2: The transcriptome libraries of non-angiosperm green plants used as the search space in this study. (DOC 25 kb) Additional file 3: 1KP scaffolds with significant similarity to virus RdRP protein sequences. (DOC 20 kb) Additional file 4: Alignment (Phylip format) of RdRP from viruses with dsRNA and related picorna-like viruses. (TXT 33 kb) Additional file 5: Maximum-likelihood tree (Newick format) of RdRPs from viruses of the picorna-like tribe. (TXT 5 kb) Additional file 6: Alignment (Phylip format) of RdRP from viruses with (–)ssRNA. (TXT 146 kb) Additional file 7: Maximum-likelihood tree (Newick format) of RdRPs from (-)ssRNA viruses. (TXT 12 kb) Additional file 8: Alignment (Phylip format) of RdRP from viruses in order Tymovirales. (TXT 11 kb) Additional file 9: Maximum-likelihood tree (Newick format) of RdRPs from viruses in order Tymovirales. (TXT 1 kb) Additional file 10: List of capsid proteins from plant and fungal viruses and of 1KP scaffolds matching one or more of these queries. (DOC 30 kb) Additional file 11: List of 30 K movement proteins from plant viruses used as queries in this study, and list of 1KP scaffolds matching one or more of these queries. (DOC 32 kb) Additional file 12: Maximum-likelihood phylogenetic tree (Newick format) of XRN nucleases from *Selaginella moellendorffii* and its relatives. (TXT 3 kb)

## Abbreviations

1KP: 1000 plants genome project
BLAST: Basic local alignment search tool
BLASTP: protein BLAST
BLASTX: BLAST search of protein databases with a translated nucleotide query
CDD: Conserved domains database
dbEST: NCBI expressed sequence tags database
dsRNA: Double-stranded RNA
EST: Expressed sequence tag
HMM: Hidden Markov Model
ICTV: International Committee for the Taxonomy of Viruses
LG: Le and Gascuel amino acid substitution model
MYA: Million years ago
NCBI: National Center for Biotechnology Information
NCLDV: Nucleocytoplasmic large DNA viruses
NR: NCBI non-redundant database of amino acid sequences
NT: NCBI nucleotide database
RdRP: RNA-dependent RNA polymerase
RT: Reverse transcriptase
ssRNA: Single-stranded RNA
TBLASTN: BLAST search of translated nucleotide databases using a protein query
WGS: Whole genome shotgun database

## Footnotes

1 Confusingly, the name *Japanese hornwort mosaic virus* has been proposed for a potyvirus isolated from *Cryptotaenia japonica* (*Apiaceae*; [52]). This angiosperm is unrelated to hornworts in a proper sense.
