# Supplementary material for: Changes in the composition of the RNA virome mark evolutionary transitions in green plants
Source: BMC Biol. 2016 Aug 15;14:68. doi: 10.1186/s12915-016-0288-8 (PMC4983792; doi:10.1186/s12915-016-0288-8)
Supplement: Additional file 1: — The library of viral RdRP domains used as queries in this study. (DOC 88 kb) [file 12915_2016_288_MOESM1_ESM.doc]

| **Family** | **Genus** | **Virus** | **GenPept ID of RdRP** |
| --- | --- | --- | --- |
| **dsRNA viruses** |  |  |  |
| *Amalgaviridae* | *Amalgavirus* | *Blueberry latent virus* | 308814348 |
| *Birnaviridae* | *Entomobirnavirus* | *Drosophila virus X* | 22855186 |
| *Chrysoviridae* | *Chrysovirus* | *Amasya cherry disease associated virus* | 158713826 |
| *Cystoviridae* | *Cystovirus* | *Pseudomonas virus phi6* | 20330566 |
| *Endornaviridae* | *Endornavirus* | *Bell pepper endornavirus* | 342243407 |
| *Hypoviridae* | *Hypovirus* | *Cryptonectria hypovirus 1* | 9626820 |
| *Partitiviridae* | *Alphapartitivirus* | *Beet cryptic virus 1* | 212681404 |
| *Partitiviridae* | *Gammapartitivirus* | *Fusarium solani virus 1* | 893388 |
| *Partitiviridae* | *Deltapartitivirus* | *Fig cryptic virus* | 332290647 |
| *Partitiviridae* | unclassified | *Ceratocystis polonica partitivirus* | 32331145 |
| *Partitiviridae* | unclassified | *Persimmon cryptic virus* | 389581809 |
| *Quadriviridae* | *Quadrivirus* | *Rosellinia necatrix quadrivirus 1* | 374504767 |
| *Reoviridae* | *(Sedoreovirinae) Phytoreovirus* | *Rice gall dwarf virus* | 134302822 |
| *Reoviridae* | *(Spinareovirinae) Fijivirus* | *Mal de Rio Cuarto virus* | 120407019 |
| *Reoviridae* | unclassified | *Raspberry latent virus* | 308928606 |
| *Totiviridae* | *Totivirus* | *Ustilago maydis virus H1* | 20564173 |
| *Totiviridae* | *Victorivirus* | *Magnaporte oryzae virus 1* | 54193769 |
| *Totiviridae* | Unclassified | *Drosophila melanogaster totivirus SW-2009a* | 268053725 |
| No family | Unclassified | *Fusarium graminearum dsRNA mycovirus-1* | 255612556 |
| No family | Unclassified | *Japanese holly fern mottle virus* | 256398148 |
| No family | Unclassified | *Persimmon latent virus* | 609253180 |
| **negative-strand RNA and ambisense RNA viruses** |  |  |  |
| *Arenaviridae* | *Mammarenavirus* | *Lassa mammarenavirus* | 23343514 |
| *Arenaviridae* | *Reptarenavirus* | *Aletinophid 1 reptarenavirus* | 401829636 |
| *Bunyaviridae* | *Hantavirus* | *Hantaan virus* | 38371717 |
| *Bunyaviridae* | *Nairovirus* | *Crimean-Congo hemorrhagic fever virus* | 76364066 |
| *Bunyaviridae* | *Orthobunyavirus* | *Bunyamvera virus* | 9630657 |
| *Bunyaviridae* | *Phlebovirus* | *Punta Toro virus* | 827882938 |
| *Bunyaviridae* | *Tospovirus* | *Groundnut bud necrosis virus* | 20153366 |
| *Bornaviridae* | *Bornavirus* | *Borna disease virus* | 306960191 |
| *Filoviridae* | *Ebolavirus* | *Zaire ebolavirus* | 10313999 |
| *Paramyxoviridae* | *Aquaparamyxovirus* | *Atlantic salmon paramyxovirus* | 700074805 |
| *Rhabdoviridae* | *Cytorhabdovirus* | *Persimmon virus A* | 523587722 |
| *Rhabdoviridae* | *Nucleorhabdovirus* | *Sonchus yellow net virus* | 9627727 |
| *Ophioviridae* | *Ophiovirus* | *Citrus psorosis virus* | 52630360 |
| *Orthomyxoviridae* | *Influenzavirus A* | *Influenza A virus* | 758899362 |
| No family | *Tenuivirus* | *Rice stripe virus* | 20428590 |
| No family | *Varicosavirus* | *Lettuce big-vein associated virus* | 212726667 |
| No family | unclassified | *Orchid fleck virus* | 149944279 |
| **positive-strand RNA viruses** |  |  |  |
| *Alphatetraviridae* | *Betatetravirus* | *Nudariella capensis beta virus* | 9631280 |
| *Barnaviridae* | *Barnavirus* | *Mushroom bacilliform virus* | 448705473 |
| *Benyviridae* | *Benyvirus* | *Beet necrotic yellow vein* | 19919908 |
| *Bromoviridae* | *Bromovirus* | *Brome mosaic virus* | 9626934 |
| *Carmotetraviridae* | *Alphacarmoteravirus* | *Providence virus* | 295984042 |
| No family | *Cilevirus* | *Citrus leprosis virus C* | 109255261 |
| *Closteroviridae* | *Closterovirus* | *Beet yellows virus* | 162416197 |
| *Closteroviridae* | unclassified closterovirus | *Rose leaf rosette-associated virus* | 686983182 |
| *Closteroviridae* | unclassified velarivirus | *Areca palm velarivirus* | 827027524 |
| *Flaviviridae* | *Flavivirus* | *Dengue virus 4* | 119390884 |
| *Flaviviridae* | unclassified | *Soybean cyst nematode virus 5* | 635260759 |
| *Hepeviridae* | *Hepevirus* | *Hepatitis E virus* | 9626448 |
| No family | *Higrevirus* | *Hibiscus green spot virus* | 355348566 |
| No family | *Idaeovirus* | *Raspberry bushy dwarf virus* | 20386797 |
| *Leviviridae* | *Allolevivirus* | *Qbeta virus* | 9630321 |
| *Luteoviridae* | *Luteovirus* | *Barley yellow dwarf-PAV virus* | 30187599 |
| *Luteoviridae* | *Polerovirus* | *Potato leaf roll virus* | 445110392 |
| *Luteoviridae* | unclassified | *Citrus vein enation virus* | 514361116 |
| *Narnaviridae* | *Mitovirus* | *Ophiostoma mitovirus 6* | 22417461 |
| *Nidovirales/Coronaviridae* | *Bafinivirus* | *White bream virus* | 116536737 |
| *Nidovirales/Mesoniviridae* | *Alphamesonivirus* | *Nam Dinh virus* | 341926385 |
| *Nodaviridae* | *Alphanodavirus* | *Flock house virus* | 22681056 |
| No family | *Ourmiavirus* | *Ourmia melon virus* | 194351518 |
| *Permutotetraviridae* | *Alphapermutotetravirus* | *Euprosterna elaeasa virus* | 18875446 |
| *Picornavirales/Dicistroviridae* | *Cripavirus* | *Drosophila virus C* | 9629651 |
| *Picornavirales* | unclassified | *Lychnis mottle virus* | 852036753 |
| *Potyviridae* | unclassified potyviridae | *Rose yellow mosaic virus* | 410493748 |
| *Secoviridae* | *Comovirus* | *Radish mosaic virus* | 188543137 |
| *Potyviridae* | *Potyvirus* | *Turnip mosaic virus* | 56961706 |
|  | *Sobemovirus* | *Turnip rosette virus* | 564293603 |
| *Togaviridae* | *Rubivirus* | *Rubella virus* | 336284683 |
| *Tombusviridae* | *Carmovirus* | *Carnation mottle virus* | 306526244 |
| *Tombusviridae* | *Umbravirus* | *Carrot mottle* | 211774537 |
| *Tymovirales/Alphaflexiviridae* | *Potexvirus* | *Potato virus X* | 215481431 |
| *Tymovirales/Tymoviridae* | *Marafivirus* | *Olive latent virus 3* | 289522103 |
| *Tymovirales/Betaflexiviridae* | *Capillovirus* | *Apple stem grooving virus* | 9629174 |
| *Tymovirales/Gammaflexiviridae* | *Mycoflexivirus* | *Botrytis virus F* | 11125723 |
| *Virgaviridae* | *Furovirus* | *Soil-borne wheat mosaic virus* | 9632355 |
| *Virgaviridae* | *Tobamovirus* | *Tobacco mosaic virus* | 19263361 |
| No family | unclassified | *Donkey orchid symptomless virus* | 559797736 |
| No family | unclassified | *Oyster mushroom spherical virus* | 28261418 |
| No family | unclassified | *Tomato chocolate spot virus* | 254733074 |

**Additional file 1.** The library of viral RdRP domains used as queries in this study.
