# Supplementary material for: Changes in the composition of the RNA virome mark evolutionary transitions in green plants
Source: BMC Biol. 2016 Aug 15;14:68. doi: 10.1186/s12915-016-0288-8 (PMC4983792; doi:10.1186/s12915-016-0288-8)
Supplement: Additional file 2: — The transcriptome libraries of non-angiosperm green plants used as the search space in this study. (DOC 25 kb) [file 12915_2016_288_MOESM2_ESM.doc]

AEKF-Penium_margaritaceum

AZZW-Chlorokybus_atmophyticus

BFIK-Entransia_fimbriata

BTFM-Monomastix_opisthostigma

CAPN-Equisetum_diffusum

CHAR-Chara_vulgaris

CYCA-Cycas_rumphii

DRGY-Chaetosphaeridium_globosum

DXOU-Nothoceros_aenigmaticus

EEMJ-Thuidium_delicatulum

EGLZ-Prumnopitys_andina

FFGR-Netrium_digitus

FFPD-Ceratodon_purpureus

FQLP-Klebsormidium_subtile

GANB-Alsophila_spinulosa

GAON-Huperzia_squarrosa

GGEA-Cedrus_libani

GINK-Ginkgo_biloba

GOWD-Sphagnum_lescurii

GTHK-Gnetum_montanum

HAOX-Spirogyra_sp

HERT-Sphaerocarpos_texanus

ISGT-Uronema_sp

ISIM-Nephroselmis_pyriformis

JADL-Rhynchostegium_serrulatum

JMXW-Bryum_argenteum

JOJQ-Cylindrocystis_cushleckae

JPYU-Marchantia_polymorpha

KYIO-Mesostigma_viride

NHCM-Angiopteris_evecta

NNHQ-Spirotaenia_minuta

NRWZ-Metzgeria_crassipilis

OPHI-Ophioglossum_petiolatum

OPHI-Ophioglossum_vulgatum

OUOI-Cunninghamia_lanceolata

PINU-Pinus_taeda

PTER-Pteridium_aquilinum

QMWB-Anomodon_attenuatus

QPDY-Coleochaete_irregularis

QVMR-Psilotum_nudum

STKJ-Cosmarium_ochthodes

SZYG-Polytrichum_commune

TCBC-Nothoceros_vincentianus

TFYI-Marchantia_emarginata

TNAW-Pyramimonas_parkeae

UPMJ-Pseudolycopodiella_caroliniana

VDAO-Ephedra_sinica

VQBJ-Coleochaete_scutata

WDCW-Mesotaenium_endlicherianum

WELW-Welwitschia_mirabilis

WJLO-Ricciocarpos_natans

WWSS-Taxus_baccata

WZYK-Bazzania_trilobata

XMGP-Juniperus_scopulorum

XNXF-Dendrolycopodium_obscurum

XRTZ-Roya_obtusa

XWHK-Rosulabryum_cf_capillare

XZUY-Cycas_micholitzii

YFZK-Sciadopitys_verticillata

YOXI-Cylindrocystis_brebissonii

YWNF-Hedwigia_ciliata

ZACW-Leucodon_brachypus

ZAMI-Zamia_vazquezii

ZRMT-Mougeotia_sp

ZZOL-Selaginella_moellendorffii_1kp

ZZOL-Selaginella_stauntoniana

**Additional file 2.** The transcriptome libraries of non-angiosperm green plants used as the search space in this study.
