# Supplementary material for: Changes in the composition of the RNA virome mark evolutionary transitions in green plants
Source: BMC Biol. 2016 Aug 15;14:68. doi: 10.1186/s12915-016-0288-8 (PMC4983792; doi:10.1186/s12915-016-0288-8)
Supplement: Additional file 3: — 1KP scaffolds with significant similarity to virus RdRP protein sequences. (DOC 20 kb) [file 12915_2016_288_MOESM3_ESM.doc]

**Contig/scaffold;Reverse match (BLASTX);Scaffold length;match length;BLAST HSP**

**Identity**

AEKF-0066821;BAB63954.1;619;191;46/191 (24%)

CAPN-1001302;CAJ32468.1;2090;229;107/229 (46%)

DRGY-0037099;NP_620728.1;5220;386;97/386 (25%)

DXOU-1060199;YP_008327312.1;387;106;29/106 (27%)

EGLZ-0032735;AAP74192.1;238;79;53/79 (67%)

GAON-0005592;CBZ05551.1;264;87;42/87 (48%)

GAON-0048667;CBZ05552.1;194;62;36/62 (58%)

GAON-0050939;CBZ05553.1;219;72;44/72 (61%)

GAON-0051485;CBZ05553.1;227;35;19/35 (54%)

GOWD-1164481;AIM55450.1;238;76;29/76 (38%)

GOWD-1205677;YP_003104770.1;481;104;34/104 (32%)

GOWD-1206285;AHH54175.1;489;79;23/79 (29%)

GTHK-0001158;YP_004936170.1;206;62;27/62 (43%)

GTHK-0001335;YP_004936170.1;206;62;27/62 (43%)

GTHK-0004810;AAR14150.1;326;102;39/102 (38%)

GTHK-0018893;YP_004464920.1;607;202;156/202 (77%)

GTHK-0033387;CBX24357.1;114;38;29/38(76%)

GTHK-0045152;AAX18600.1;151;49;26/49 (53%)

GTHK-0057625;ACJ04421.1;257;60;30/60 (50%)

GTHK-0057841;AGT55876.1;259;55;28/55 (50%)

GTHK-0066193;AFK73722.1;537;167;112/167 (67%)

GTHK-0067580;AFU07531.1;683;216;101/216 (47%)

GTHK-0070069;CAC10493.2;1861;538;295/538 (54%)

HAOX-0002392;NP_624325.1;1341;110;31/110 (28%)

HAOX-0006897;AIE77246.1;2878;181;68/181 (37%)

HAOX-0013050;CBZ05553.1;113;33;23/33 (69%)

HAOX-0018793;AHF48622.1;201;66;35/66 (53%)

HAOX-0020277;AAY51483.1;245;79;39/79 (49%)

HAOX-0021062;AAY51483.1;270;99;55/99 (55%)

HAOX-0026100;AIE77246.1;3422;244;64/244 (26%)

JADL-0003618;AJG39259.1;3680;88;29/88 (32%)

JADL-0014334;BAM13786.1;1643;204;53/204 (25%)

JADL-0015513;CCV01186.1;2235;361;104/361 (28%)

JADL-0049413;AIZ49763.1;340;77;22/77 (28%)

JADL-0050886;AHE13863.1;380;111;35/111 (31%)

JADL-0057482;AJG39259.1;758;133;40/133 (30%)

JADL-0062324;CEJ20912.1;2785;337;83/337 (24%)

JADL-0062521;AFY52607.1;4436;718;157/718 (21%)

JADL-0062527;BAM13786.1;5002;354;94/354 (26%)

JADL-0062531;AJG39263.1;5464;875;174/875 (19%)

JOJQ-0005112;AIE77246.1;698;197;61/197 (30%)

JPYU-0008008;AIS39947.1;480;118;46/118 (38%)

JPYU-0051856;AGW51759.1;680;144;45/144 (31%)

NRWZ-0131294;AFX73022.1;373;115;57/115 (49%)

Pinus_taeda4863;AFI24690.1;358;107;56/107 (52%)

QMWB-0068547;AII01815.1;266;76;25/76 (32%)

QMWB-0087057;CAB65142.1;765;128;38/128 (29%)

QPDY-0003073;ACA61232.1;396;142;74/142 (52%)

QPDY-0006608;ACA61232.1;332;60;39/60 (65%)

QPDY-0009355;YP_007697651.1;4648;599;229/599 (38%)

QPDY-0010402;YP_001497151.1;930;185;111/185 (60%)

QPDY-0011478;YP_001497151.1;1284;444;205/444 (46%)

QPDY-0012300;AFH09414.1;4652;779;321/779 (41%)

QPDY-0018370;ADQ54106.1;123;39;31/39 (79%)

QPDY-0034458;YP_001497151.1;282;93;62/93 (66%)

QPDY-0035095;ACA61232.1;293;95;59/95 (62%)

QPDY-0042233;YP_001497151.1;544;179;85/179 (47%)

QPDY-0049591;YP_001497151.1;2289;377;159/377 (42%)

SGTW-0036589;AAY51483.1;236;78;53/78 (67%)

SGTW-0046943;AAY51483.1;844;248;173/248 (69%)

TCBC-0020112;AHC70111.2;1625;187;53/187 (28%)

TCBC-0022728;NP_047560.1;260;59;27/59 (45%)

TCBC-0022921;AFX73019.1;270;92;54/92 (58%)

TCBC-0129656;ADV15444.1;453;150;117/150 (78%)

TCBC-0131519;ADV15444.1;520;173;104/173 (60%)

TCBC-0135701;AFQ95555.1;912;301;212/301 (70%)

TFYI-0089137;AGJ03719.1;664;238;59/238 (24%)

TNAW-0096324;NP_660180.1;1498;438;122/438 (27%)

TNAW-0096738;AIE77246.1;1724;110;40/110 (36%)

UPMJ-0003822;NP_853560.2;680;86;32/86 (37%)

UPMJ-0005369;NP_848527.1;931;202;81/202 (40%)

UPMJ-0010308;AIF28241.1;228;31;22/31 (70%)

UPMJ-0017793;NP_848527.1;775;95;50/95 (52%)

UPMJ-0028709;AAK69629.2;965;194;87/194 (44%)

UPMJ-0107208;AIF28241.1;334;111;63/111 (56%)

UPMJ-0108463;AIF28241.1;353;117;40/117 (34%)

UPMJ-0115952;CAA67042.1;528;131;51/131 (38%)

WZYK-0032216;AHL25284.1;1950;138;46/138(33%)

WZYK-0079471;AIF71030.1;201;46;21/46 (46%)

WZYK-0119611;161088126;908;284;69/284 (24%)

XMGP-0053972;YP_001274391.1;342;109;67/109 (61%)

YFZK-0007255;AFA36170.1;1305;394;202/394 (51%)

YFZK-0009646;AFA36170.1;1625;441;162/441 (36%)

YFZK-0011044;YP_009130617.1;6092;349;107/349 (30%)

YWNF-0001895;AHE13863.1;1339;224;63/224 (28%)

YWNF-0014965;AHE13863.1;5902;513;124/513 (24%)

YWNF-0016114;AKC89288.1;2115;453;96/453 (21%)

YWNF-0016602;CCP46989.1;1907;268;70/268 (26%)

YWNF-0076309;CCP46989.1;937;284;72/284 (25%)

YWNF-0080910;YP_006590079.1;2330;233;58/233 (24%)

YWNF-0081168;AJG39263.1;2763;433;106/433 (24%)

ZACW-0164371;AAX63415.1;466;155;123/155 (79%)

ZACW-0166088;AHG97565.1;498;166;136/166 (81%)

ZZOL-0105930;AHC70111.2;863;96;28/96 (29%)
