# Supplementary material for: Changes in the composition of the RNA virome mark evolutionary transitions in green plants
Source: BMC Biol. 2016 Aug 15;14:68. doi: 10.1186/s12915-016-0288-8 (PMC4983792; doi:10.1186/s12915-016-0288-8)
Supplement: Additional file 10: — List of capsid proteins from plant and fungal viruses and of 1KP scaffolds matching one or more of these queries. (DOC 30 kb) [file 12915_2016_288_MOESM10_ESM.doc]

gi|641481249|ref|YP_009032648.1| capsid protein [Carnation mottle virus]

gi|215481435|ref|YP_002332933.1| coat protein [Potato virus X]

gi|289522105|ref|YP_003475891.1| coat protein [Olive latent virus 3]

gi|9632358|ref|NP_049339.1| 19 kDa capsid protein [Soil-borne wheat mosaic virus]

gi|19263363|ref|NP_597750.1| Coat protein [Tobacco mosaic virus]

gi|308814347|ref|YP_003934624.1| ORF1 [Blueberry latent virus]

gi|169793710|gb|ACA81390.1| coat protein [Beet cryptic virus 1]

gi|332290649|ref|YP_004429259.1| putative capsid protein [Fig cryptic virus]

gi|389581807|ref|YP_006390090.1| coat protein [Persimmon cryptic virus]

gi|134305392|ref|NP_042281.2| nucleocapsid protein [Sonchus yellow net virus]

gi|52630365|ref|YP_089664.1| 49 kDa protein [Citrus psorosis virus]

| **Query** | **1KP match, score in bits, and E value** |
| --- | --- |
| gi|289522105|ref|YP_003475891.1| coat protein [Olive latent virus 3] | scaffold-GTHK-0070069-Gnetum_montanum 177 9e-44 |
| gi|332290649|ref|YP_004429259.1| putative capsid protein [Fig cryptic virus] | scaffold-GAON-0024525-Huperzia_squarrosa 150 2e-35 |
| gi|52630365|ref|YP_089664.1| 49 kDa protein [Citrus psorosis virus] | scaffold-UPMJ-0117727-Pseudolycopodiella_caroliniana 105 9e-22 |
| gi|169793710|gb|ACA81390.1| coat protein [Beet cryptic virus 1] (489 letters) | scaffold-TCBC-0136809-Nothoceros_vincentianus 87 2e-16 |
| gi|169793710|gb|ACA81390.1| coat protein [Beet cryptic virus 1] (489 letters) | scaffold-EGLZ-0044553-Prumnopitys_andina 55 8e-07 |

**Additional file 6.** List of capsid proteins from plant and fungal viruses and of 1KP scaffolds matching one or more of these queries.
