# Supplementary material for: Changes in the composition of the RNA virome mark evolutionary transitions in green plants
Source: BMC Biol. 2016 Aug 15;14:68. doi: 10.1186/s12915-016-0288-8 (PMC4983792; doi:10.1186/s12915-016-0288-8)
Supplement: Additional file 11: — List of 30 K movement proteins from plant viruses used as queries in this study, and list of 1KP scaffolds matching one or more of these queries. (DOC 32 kb) [file 12915_2016_288_MOESM11_ESM.doc]

211774535_Umbra

25013720_Nepo

20889383_Citri

25013766_Sequi

20386799_Idaeo

544395672_Badna

9626929_Alfamo

9626207_Tricho

25013922_Sadwa

126010929_Torrado

9626939_Caulimo

194351520_Ourmia

9790332_Tombus

339779242_Tospo

20429234_Diantho

9632356_Furo

9626473_Cucumo

641467690_Aureus

52630363_Ophio

25013704_Como

326910949_Emara

52137002_Chera

83659774_Cytorhabdo

357579589_Viti

20889308_Begomo

109255270_Cile

19263362_Tobamo

| **Query** | **1KP match, score in bits, and E value** |
| --- | --- |
| 544395672 | scaffold-UPMJ-0124711-Pseudolycopodiella_caroliniana 178 3e-44  scaffold-UPMJ-0124564-Pseudolycopodiella_caroliniana 172 2e-42  scaffold-UPMJ-0015359-Pseudolycopodiella_caroliniana 167 5e-41  scaffold-UPMJ-0028690-Pseudolycopodiella_caroliniana 159 3e-38  scaffold-UPMJ-0015715-Pseudolycopodiella_caroliniana 100 7e-21  scaffold-UPMJ-0005779-Pseudolycopodiella_caroliniana 94 1e-18 |
| 544395672 | Pinus_taeda58374 55 5e-07  Pinus_taeda54739 52 4e-06 |
| 9626207 | Pinus_taeda54739 56 2e-07  Pinus_taeda58374 54 1e-06 |
| 9626207 | scaffold-YFZK-0015786-Sciadopitys_verticillata 55 7e-07 |
| 9626939 | Pinus_taeda58374 54 1e-06 |
| 9626939 | scaffold-YFZK-0015786-Sciadopitys_verticillata 47 2e-04 |
| 52630363 | scaffold-UPMJ-0124384-Pseudolycopodiella_caroliniana 71 7e-12 |
| 83659774 | Pinus_taeda28696 41 0.010 (?) |
| 83659774 | Pteridium_aquilinum_Contig19990 40 0.013 (?) |

**Additional file 7.** List of 30K movement proteins from plant viruses used as queries in this study, and list of 1KP scaffolds matching one or more of these queries.
